# Case Report: Hypodipsic hypernatremia secondary to hydrocephalus in a dog

**DOI:** 10.3389/fvets.2025.1579965

**Published:** 2025-05-16

**Authors:** Maddisen W. Antes, Amelia C. Corona, Helena Rylander

**Affiliations:** University of Wisconsin-Madison, Madison, WI, United States

**Keywords:** hypernatremia, hypodipsia, hydrocephalus, mentation, dog

## Abstract

A 6-year-old male castrated golden retriever was presented for inappetence, lethargy, and progressive obtundation. A chemistry panel showed hypernatremia (>190 mmol/L) and hyperchloremia (157 mmol/L). Brain magnetic resonance imaging (MRI) showed severe congenital hydrocephalus with compression of major brain structures, including marked thinning of gray and white matter of the prosencephalon. Results of cerebrospinal fluid (CSF) analysis were within the reference range. A hypodipsic hypernatremia was suspected secondary to dysregulation of the osmoreceptors controlling the thirst center due to progression of hydrocephalus. The dog was treated with intravenous (IV) fluids to slowly reduce the hypernatremia over several days. Mental status improved with normalization of the blood sodium level. Increased stress due to a long car ride and a change in routine may have been the catalyst for the hypodipsia. Hypodipsic hypernatremia is a rare complication of hydrocephalus and should be on the differential list when a sudden change in mentation occurs in dogs with hydrocephalus.

## Introduction

Hypernatremia in dogs is characterized by a sodium concentration of greater than 155 mmol/L and may be caused by ingestion or administration of excess sodium, or by a pure water deficit due to loss of hypotonic fluids, or reduced intake of water (1). Hypodipsia can occur secondary to neurologic dysfunction or may occur due to the lack of access to water (1, 2).

Hypernatremia secondary to a free water deficit leads to a hypertonic hyperosmolar state, known as hypertonic dehydration (2). Clinical signs associated with a hypertonic dehydration include weakness, irritability, and ataxia that may rapidly develop to seizures and stupor or coma as the hypernatremia worsens.

In this case report, we describe a case of hypodipsic hypernatremia secondary to congenital hydrocephalus in a dog.

## Case description

**Figure 1:**
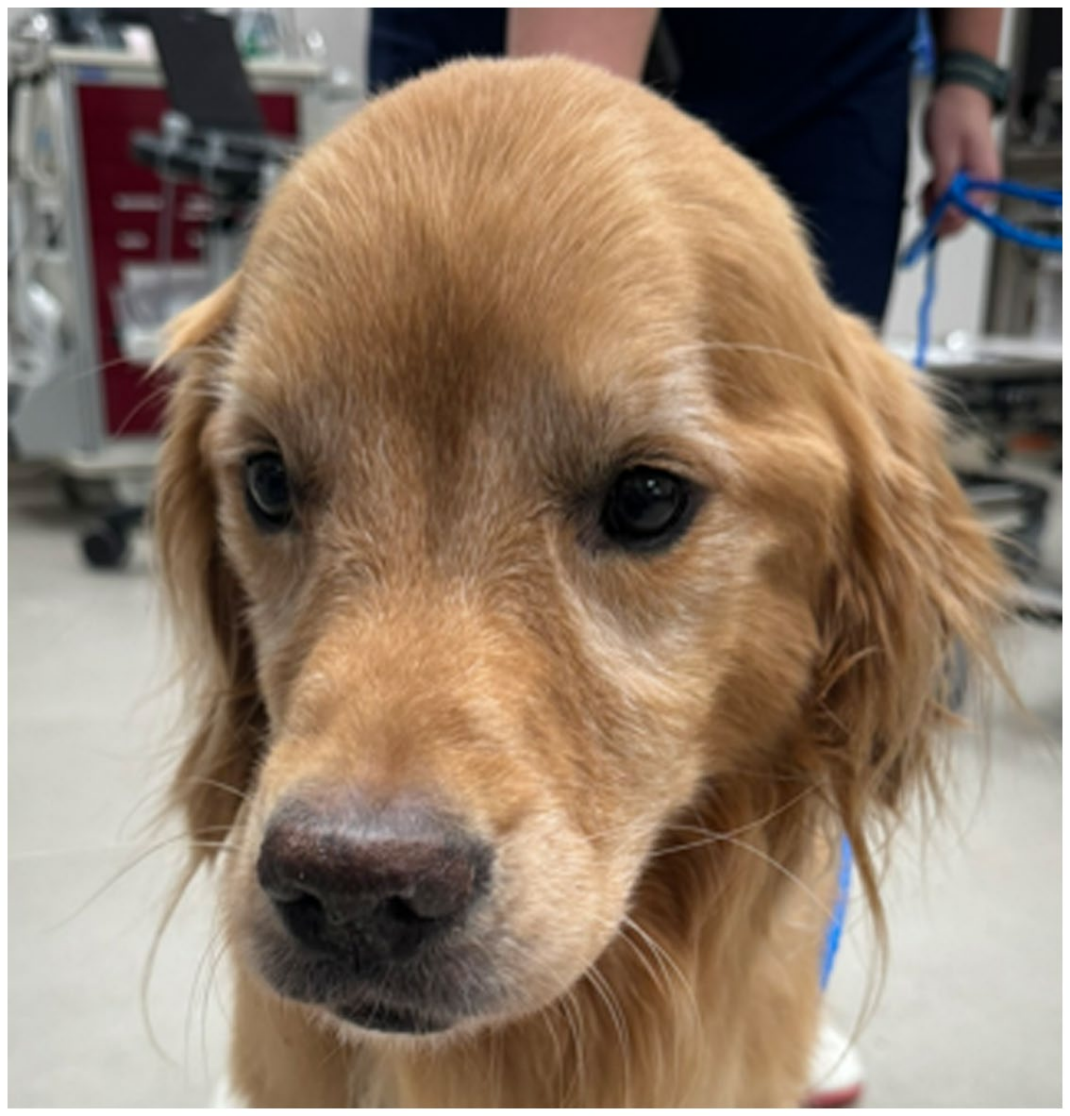
A 6-year-old golden retriever with a dome-shaped head. Note the masticatory muscle atrophy.

**Figure 2:**
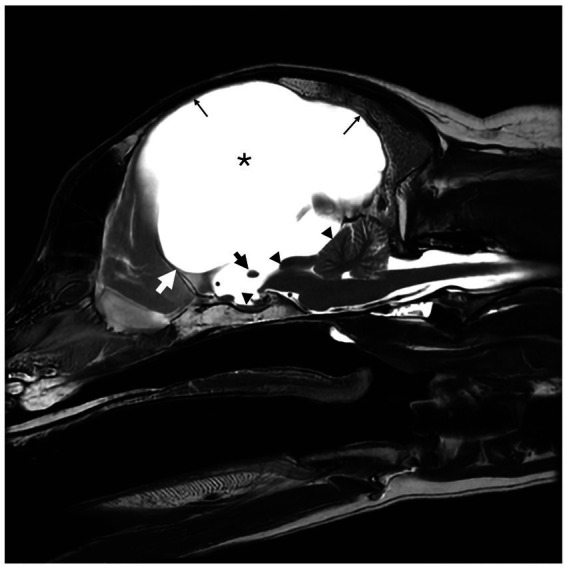
T2-weighted (T2W) sagittal magnetic resonance image (MRI) of a 6-year-old male neutered golden retriever. Marked hydrocephalus with thinning of the cerebrum secondary to ventriculomegaly is noted by (*). The small black arrow notes the markedly thinned interthalamic adhesion. The small white arrow delineates the rostral aspect of the third ventricle, at the level of the lamina terminalis. The corpus callosum is absent. The mesencephalic aqueduct is markedly narrowed with secondary rostral flattening of the cerebellum and compression of the pituitary gland (small black triangles). There is diffuse thinning of the cerebral cortex (thin black arrow).

**Figure 3:**
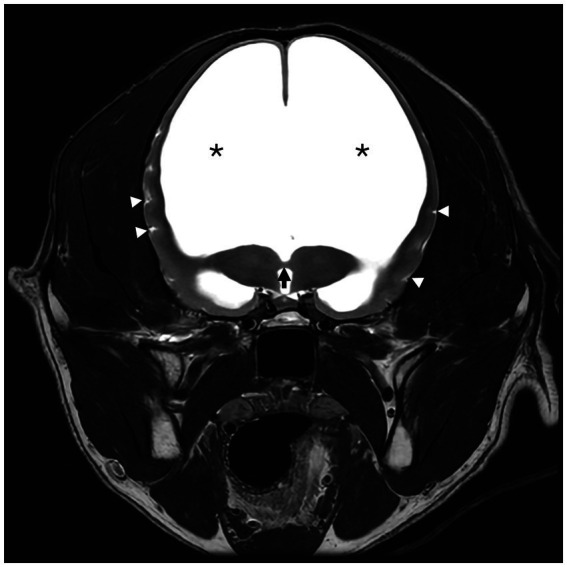
T2-weighted (T2W) transverse magnetic resonance image (MRI) of a 6-year-old male neutered golden retriever. The interthalamic adhesion is markedly thinned (black arrow). The septum pellucidum and corpus callosum are absent. Marked lateral ventriculomegaly is denoted by (*). There is decreased CSF signal within the sulci of the remaining thin cerebral cortex (white triangles).

A 6-year-old male castrated golden retriever dog, weighing 23.7 kg, presented for a 2-week history of inappetence, lethargy, and progressive obtundation. He had been traveling with the owners by car across the country for the past 2 weeks when the signs occurred. Previous history was suspected congenital hydrocephalus [due to his dome shaped head (Figure 1) and epileptic seizures], hypothyroidism [controlled with levothyroxine 0.03 mg/kg per os (PO) q 12 h], inflammatory bowel disease, allergic dermatitis both controlled with prednisone (0.42 mg/kg q 24 h) and seizures, which had increased in frequency. His first generalized epileptic seizure occurred at 6 months of age, at which time he was started on levetiracetam (21 mg/kg q 8 h). He had two more generalized epileptic seizures over the next 5 years. Two months prior to presentation, he had a generalized epileptic seizure, and the levetiracetam was changed to extended release (42 mg/kg q 12 h). He had another similar seizure 2 days prior to presentation. Abnormal findings on physical examination were as follows: a body condition score of 2 of 9, a muscle condition score 1 of 3, a dome-shaped head (Figure 1), and reduced range of motion in the right tarsal joint caused by an unknown injury as a puppy. The body temperature was normal (38.7°C). Neurologic examination revealed moderate obtundation, a vestibular ataxia with a wide-based stance, circling to the right, absent menace response in both eyes (OU), inducible vertical to rotary nystagmus in lateral and dorsal recumbency, positional ventrolateral strabismus OU, and absent paw placement in all limbs. The neuroanatomic localization was multifocal brain involving the prosencephalon and brainstem. Differential diagnoses were anomalous (worsening of suspected hydrocephalus), infection, inflammation, or neoplasia. A complete blood count (CBC) showed a normal white blood cell count (13,800; *N* = 500–14,000). A chemistry panel showed hypernatremia (>190 mmol/L; *N* = 141–158 mmol/L) and hyperchloremia (157 mmol/L, *N* = 110–119 mmol/L). Urine specific gravity (USG) was 1.068 (*N* = 1.015–1.060). Thoracic radiographs were within normal limits. The dog was anesthetized for magnetic resonance imaging (MRI) of the brain (GE Healthcare, 1.5 Tesla, Milwaukee, Wisconsin). He was premedicated with fentanyl (4.2 μg/kg) and lidocaine (2.1 mg/kg), and induced with propofol (1.7 mg/kg) IV and maintained on sevoflurane and oxygen. The systolic blood pressure was 120 mmHg at induction, went down to 80 mmHg, and then returned to 120 mmHg at the end of the anesthesia. The dog was receiving Plasmalyte with D5W and Plasmalyte with KCl IV during the anesthesia. The MR images obtained of the brain were T2-weighted (T2W) sagittal and transverse, T1-weighted (T1W) pre- and post-contrast (gadolinium) sagittal, transverse and dorsal, fluid attenuation inversed recovery (FLAIR) sagittal. The MRI showed marked dilation of the lateral ventricles with a small thalamus, compression of the pituitary gland, markedly narrowed mesencephalic aqueduct, marked thinning of gray and white matter of the cerebrum, and flattening of the rostral aspect of the cerebellum (Figures 2, 3). There was no contrast enhancement. These findings were compatible with congenital hydrocephalus. Cerebrospinal fluid (CSF) was collected from the cerebellomedullary cistern without any complications. Results of cerebrospinal fluid analysis (total nucleated cell count, differential count, and protein content) were within reference range. A central line was placed before the patient recovered.

A hypodipsic hypernatremia was suspected secondary to dysregulation of the osmoreceptors controlling the thirst center due to progression of the congenital hydrocephalus.

**Figure 4:**
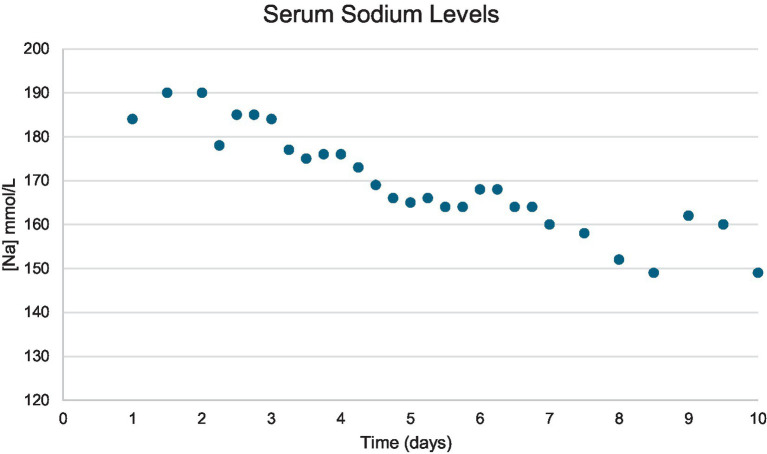
Graph showing the serum sodium level content change over time in a 6-year-old male neutered golden retriever.

The dog was hospitalized, and IV fluid therapy was started to reduce the high serum sodium content. The initial fluid rate was 40.5 mL/kg/day plasmalyte with 20 mEq/L potassium, 30.4 mL/kg/day dextrose 5% in water (D5W), and the dog did not have access to free water. Serum sodium levels were checked every 6–8 h, and the fluid rate was adjusted to maintain hydration and reduce the serum sodium content at a safe rate of 0.5 mEq/L/h (Table 1; Figure 4). The dog progressively became brighter as his sodium normalized, and he remained neurologically stable. The USG on day 3 was >1.050. A nasogastric (NG) tube was placed after 6 days to provide enteral plain water, which helped with the regulation of serum sodium.

**Table 1:** The serum sodium levels and rate of fluid in a 6-year-old male neutered golden retriever.

Day Hospitalization /Time	D1 T0	D1 17:00	D1 23:43	D2 00:00	D2 08:00	D2 14:00	D2 20:00	D3 00:00	D3 08:00	D3 14:00	D3 20:00	D4 02:00	D4 08:00	D4 14:00	D4 19:00
Na (mmol/L)	184	> 190	> 190	>190	178	185	185	184	177	175	176	176	173	169	166
Av. IV Fluid Rate (ml/kg/day)	40.5	40.5	40.5	40.5	44.0	55.1	46.2	33	33	38.1	38.1	43.4	43.4	43.4	43.4
Av. D5W Rate (ml/kg/day)	30.4	30.4	30.4	30.40	33.0	0	0	10.3	10.3	5.2	5.2	10.3	10.3	10.3	5.2
Weight (kg)	23.7			21.8				23.3				23.2			

Day Hospitalization /Time	D5 00:00	D5 07:00	D5 14:00	D5 19:00	D6 02:00	D6 07:00	D6 19:00	D6 20:00	D7 04:00	D7 07:00	D8 11:00	D8 16:00	D9 12:00	D9 22:00	D10 20:00
Na (mmol/L)	165	166	164	164	168	168	164	164	160	158	152	149	162	160	149
IV Fluid Rate (ml/kg/day)	40.8	40.8	31.1	24.3	24.6	24.6	39.3	39.3	37.6	37.6	37.2	37.2	23.5	23.5	23.5
D5W Rate (ml/kg/day)	4.9	9.7	19.4	29.1	34.4	34.4	34.4	34.4	32.9	0	0	0	0	0	0
Free water ml/kg/day					19.7				18.8		14		9.4		9.4
Weight (kg)	24.7				24.4				25.5		25.8		25.5		25.5

D, day; Av., average; IV, intravenous; Na, sodium; D5W, dextrose 5% in water.

On day 5 of hospitalization, the central line in the right saphenous vein was removed and replaced by a central line in the left saphenous vein. On day 6 of hospitalization, a fever (39.6–40.7°C) was noted. The origin of the fever was not determined at that time.

On day 8 of hospitalization, the fever persisted (39.6°C). Pitting edema and progressive bruising were present at the level of the left stifle into the inguinal region. The central line was removed. Vascular ultrasound revealed a large thrombus in the vena cava extending into the left femoral vein, occluding up to 77% of the total diameter of the vessel. The dog was started on anti-thrombotic medication (enoxaparin 0.78 mg/kg subcutaneously every 6 h) and ampicillin/sulbactam (Unasyn; 27.9 mg/kg IV q 6 h), which 2 days later was switched to oral ampicillin/sulbactam (14.6 mg/kg q 12 h). The fever resolved 2 days later.

The dog started drinking water on day 7 of his hospitalization, and D5W was discontinued. On day 8 of hospitalization, the NG tube was replaced by an esophageal tube (E-tube) for continued plain water supplementation. Intravenous fluids were continued throughout the dog’s hospital stay.

The dog was discharged on day 10 of hospitalization. At that time, his temperature remained normal, he was eating and drinking, and his serum sodium concentration was 149 mmol/L. Neurologic examination at discharge showed marked improvement with an appropriate mentation, decreased menace response OU, and a mild pelvic limb ataxia. He was sent home with amoxicillin/clavulanic acid (14.6 mg/kg PO q 12 h) and enoxaparin (0.17 mg/kg subcutaneously q 6 h).

On recheck examination 5 days later, the patient was reportedly voluntarily eating and drinking readily and received 750 mL of plain water supplementation via the E-tube per day. Physical examination revealed mild ecchymoses on the lateral and cranial aspect of the left tarsus. Neurologic examination was unchanged. The enoxaparin was discontinued and replaced by rivaroxaban (0.84 mg/kg q 24 h r), as the thrombus in the vena cava was smaller in size but still present. The USG one week later was 1.041. The serum sodium level was normal at 157 mmol/L and 1 month later at 151 mmol/L.

The E-tube was removed 27 days after discharge.

Placement of a ventriculoperitoneal shunt was declined by the owners.

At a phone update 7 months later, the dog was still behaving normally and was drinking enough water on his own. There had been three more epileptic seizures after the visit, with the last one occurring 3 months ago.

## Discussion

Serum osmolality is tightly controlled by neural mechanisms in the lamina terminalis, the juxtaglomerular apparatus in the kidney, and the neurohypophysis (3, 4). The normal osmolality of dogs is between 290 and 310 mOsm/kg.

Lesions affecting the rostral aspect of the third ventricle in the area of the lamina terminalis have been shown to cause abnormalities in thirst regulation, with or without absence of arginine vasopressin (AVP) production (3). The lamina terminalis forms from the rostral-most aspect of the neuropore during development, and is formed from three nuclei: the subfornicular organ (SFO), the organum vasculosum (OVLT), and the median preoptic nucleus (MnPO) (3). The lamina terminalis represents the rostral-most aspect of the hypothalamus. The SFO and OVLT each are exposed to the peripheral vasculature via fenestrated capillaries (3) (outside the blood–brain barrier) and convey information about plasma osmolality and circulating levels of angiotensin II (ANG II) to the MnPO (5). The MnPO is thought to act as a relay center for signals from the SFO and OVLT (1, 3). While lesions of the SFO do appear to cause some derangements in thirst, lesions of this region do not fully prevent water intake (4). In contrast, lesions affecting the OVLT can result in a fully adipsic state (4). In our patient, the hypernatremia only developed after a stressful event and a change in routine, which suggests that there was some maintained function of water regulation.

We report on a rare case of hypodipsic hypernatremia attributable to congenital hydrocephalus.

Hypodipsic hypernatremia has been associated with various malformations in humans, cats, and dogs. In humans, it has been linked to hydrocephalus (6), hypothalamic tumors (7), various types of holoprosencephaly (8–10), and abnormal calcifications within the brain (11, 12). In cats, it has been reported in association with hydrocephalus (13), lobar holoprosencephaly (14), and head trauma (15). In dogs, it has been reported secondary to lobar holoprosencephaly in miniature schnauzers (16), dysgenesis of the corpus callosum (17–19), focal hypothalamic granulomatous meningoencephalitis (20), and glioblastoma multiforme (21). The cause of hypodipsic hypernatremia in many of these cases has been attributed to dysfunction of or dysregulation of the osmoreceptors in the lamina terminalis. In a case review including 11 dogs with hypodipsia secondary to dysgenesis of the corpus callosum, 6 were miniature schnauzers (17). In one mixed breed dog, hypodipsic hypernatremia was associated with atrophy of the septum pellucidum, neuroaxonal dystrophy of the cuneate nuclei, and moderate hydrocephalus, though it was not clear if these findings were the cause for the patient’s clinical signs (17, 22). It is likely that the cause of the hypernatremia in our patient was from a similar mechanism. Considering that many dogs with hydrocephalus are lacking major brain structures, including the corpus callosum and lamina terminalis, it is interesting that not more of these patients develop hypodipsia. However, without a necropsy, the exact functional cause of the hypodipsia in our patient cannot be determined. The hyperosmolality that occurs in hypertonic dehydration results in an osmotic gradient that promotes loss of intracellular fluid to the extracellular space. To prevent excess loss of intracellular water in the face of hypernatremia, the brain rapidly develops intracellular idiogenic osmoles, which help to regulate the osmotic gradient that is produced with hypernatremia and maintain normal intracellular water volume (23). These idiogenic osmoles may develop as soon as 1 h after hypernatremia has developed (2). Full compensation via idiogenic osmoles may take as long as 2–7 days, and allows for adaptation to a hyperosmolar state in the extracellular space (5). As the free water deficit is corrected, the osmotic gradient will reverse, and water will flow intracellularly. Rapid correction of hypernatremia can cause significant cerebral edema due to the rapid influx of water into neurons (4). For this reason, it is important to slowly correct free water deficits in these cases, usually over at least 3 days with a maximum correction of 1 mmol/L/h or 0.5 mmol/L/h in cases where the sodium exceeds 180 mmol/L (23). In our case, the hypernatremia was corrected slowly over 10 days by checking the serum sodium concentration frequently and adjusting the fluid rate accordingly. The patient improved neurologically and returned to his neurologic status before the acute hypernatremia occurred. His water consumption returned to normal, and the owners were observant of lethargy or any new neurologic signs that would suggest hypernatremia. By always making sure that the dog had access to water and drank readily, episodes of hypernatremia were avoided in future.

Since cases of hypodipsia secondary to hydrocephalus are rarely reported, it is not possible to know if a ventriculoperitoneal shunt placement would have improved the hypodipsia in our dog. The dog had mild clinical signs from the hydrocephalus until he became hypernatremic, and with the correction of the hypernatremia, without addressing the hydrocephalus, the dog returned to its previous neurologic status with very mild signs. Due to the mild signs, the owner declined surgery for ventriculoperitoneal shunt placement.

The dog in this case report presented with severe hypernatremia with normally to excessively concentrated urine. Urine concentration is dependent mainly on the production of AVP, thus suggesting that this patient likely had normal production of AVP (4). The suspected mechanism of hypodipsic hypernatremia in this case is pressure necrosis of the hypothalamus secondary to worsening of congenital hydrocephalus, leading to dysfunction of the lamina terminalis and its control over the generation of thirst sensation. It is likely that the stress due to the long car ride, new environment, and a change in routine made this dog drink less and exacerbated the dysfunction of the lamina terminalis, leading to him becoming hypernatremic, as he did not have the normal thirst drive.

## Conclusion

Hypodipsic hypernatremia, although rare, should be a differential diagnosis in dogs with hydrocephalus that have a sudden decline in mentation. A stressful event or a change in routine may be the catalyst for this condition.

## Data Availability

The original contributions presented in the study are included in the article/supplementary material, further inquiries can be directed to the corresponding author.
